# Supplementary figures and images for: Urine-Based Detection of Biomarkers Indicative of Chronic Kidney Disease in a Patient Cohort from Ghana
Source: J Pers Med. 2022 Dec 24;13(1):38. doi: 10.3390/jpm13010038 (PMC9863148; doi:10.3390/jpm13010038)

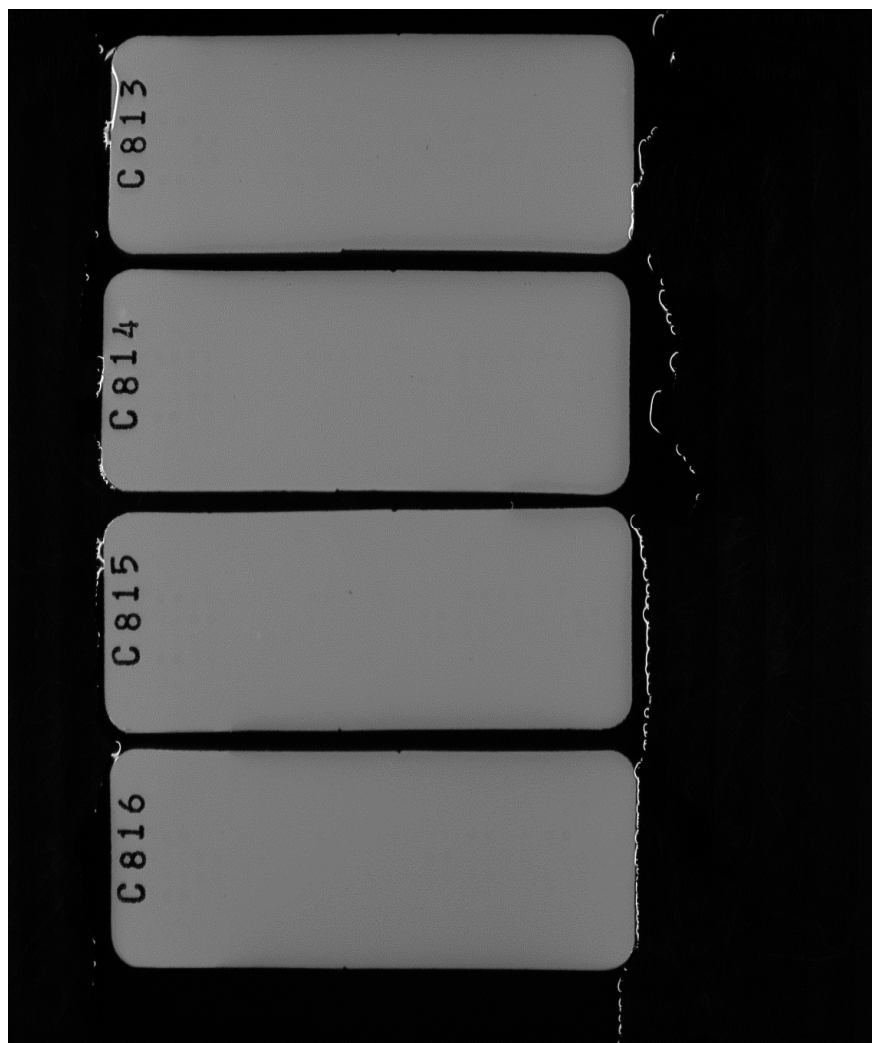

**C813: CDK male**  
**C814: CDK female**

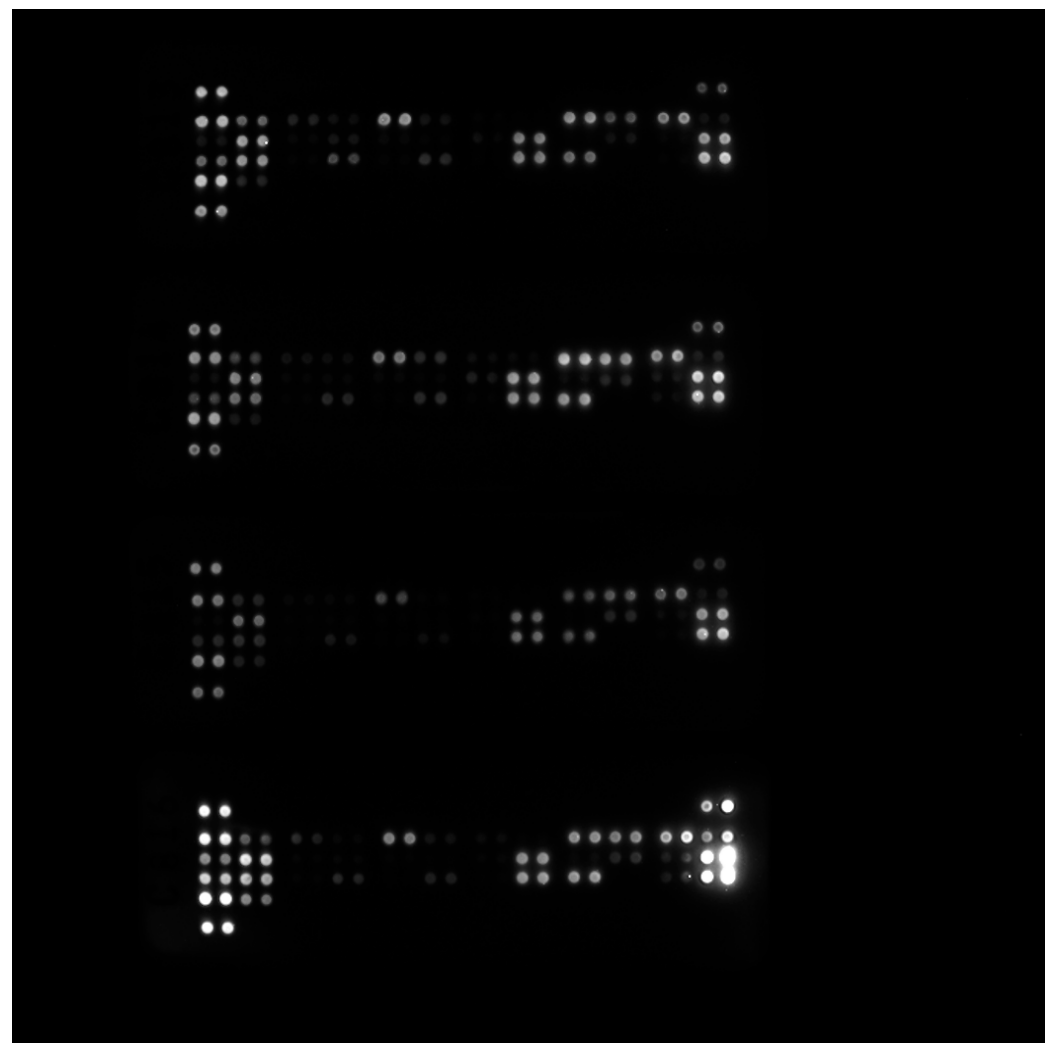

**C815: Control male**  
**C816: Control female**

Supplement: Supplementary file 1 [file jpm-13-00038-s001.zip › Figure S1.pdf]

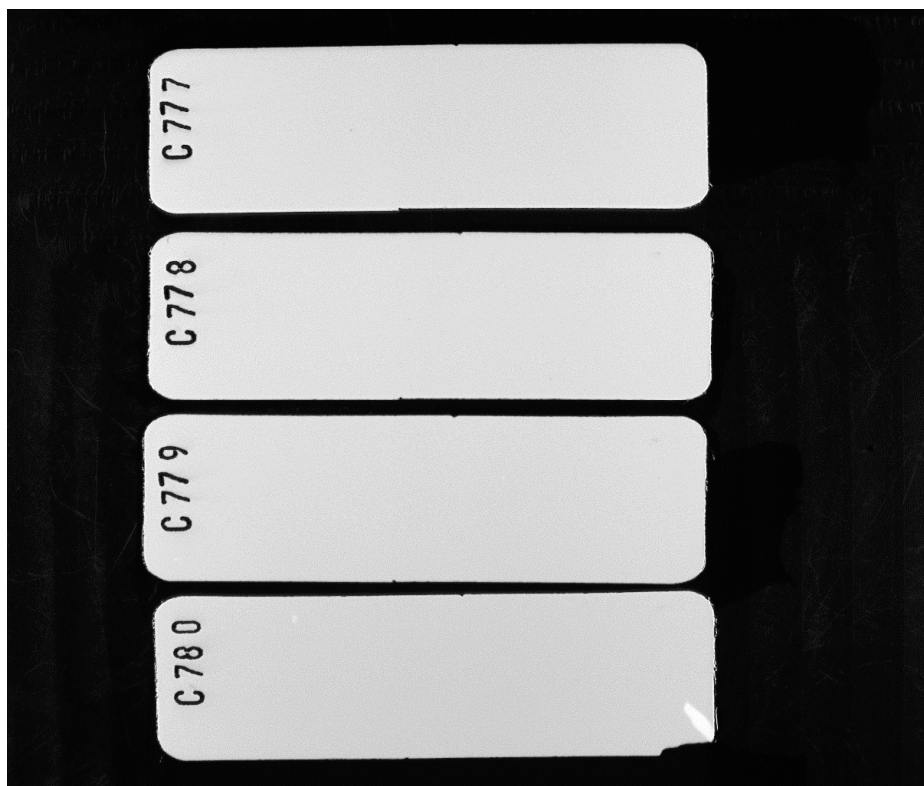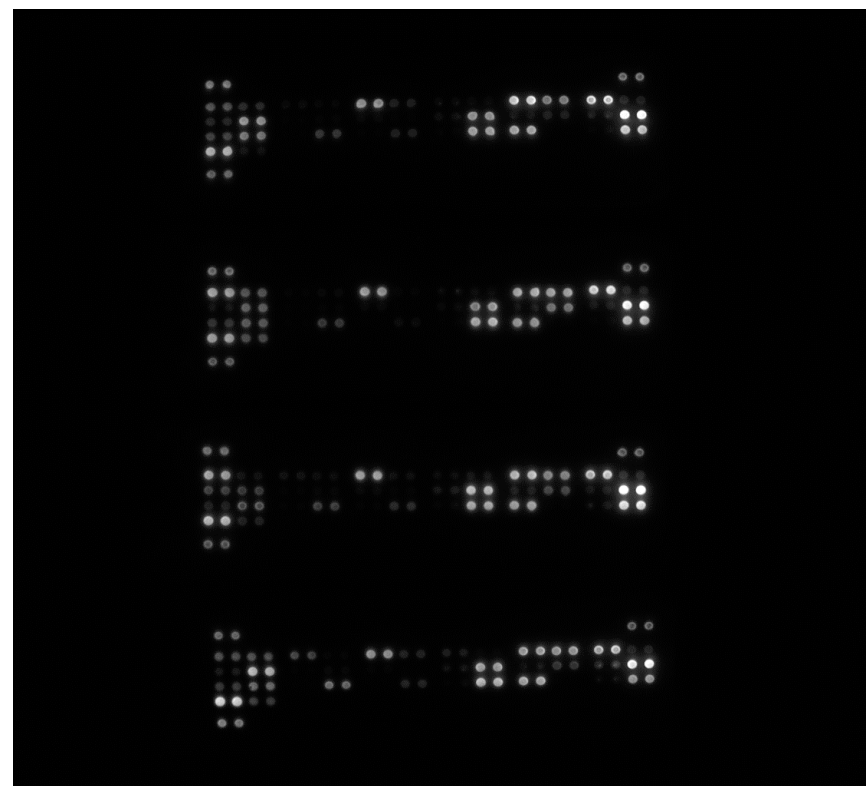

**C779: CDK male**  
**C780: CDK female**

**C777: Control male**  
**C778: Control female**

Supplement: Supplementary file 1 [file jpm-13-00038-s001.zip › Figure S2.pdf]

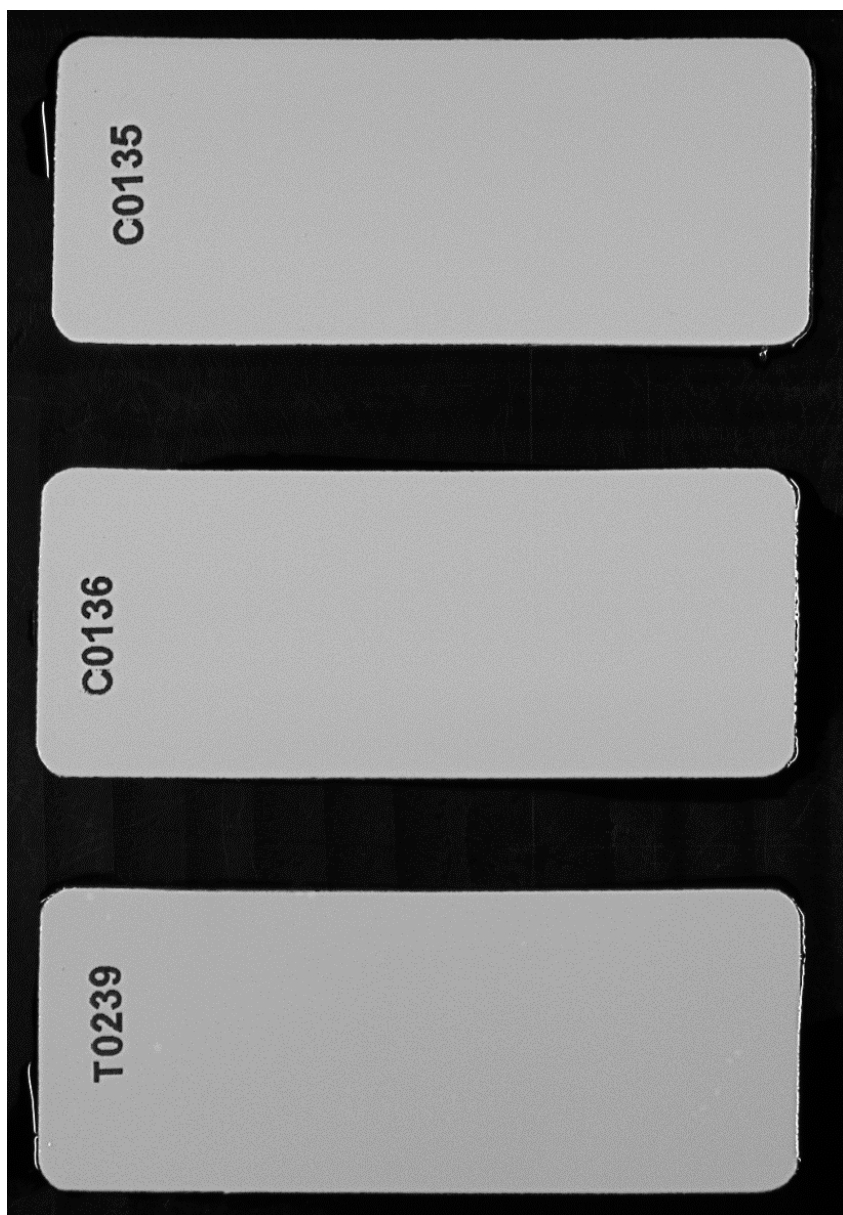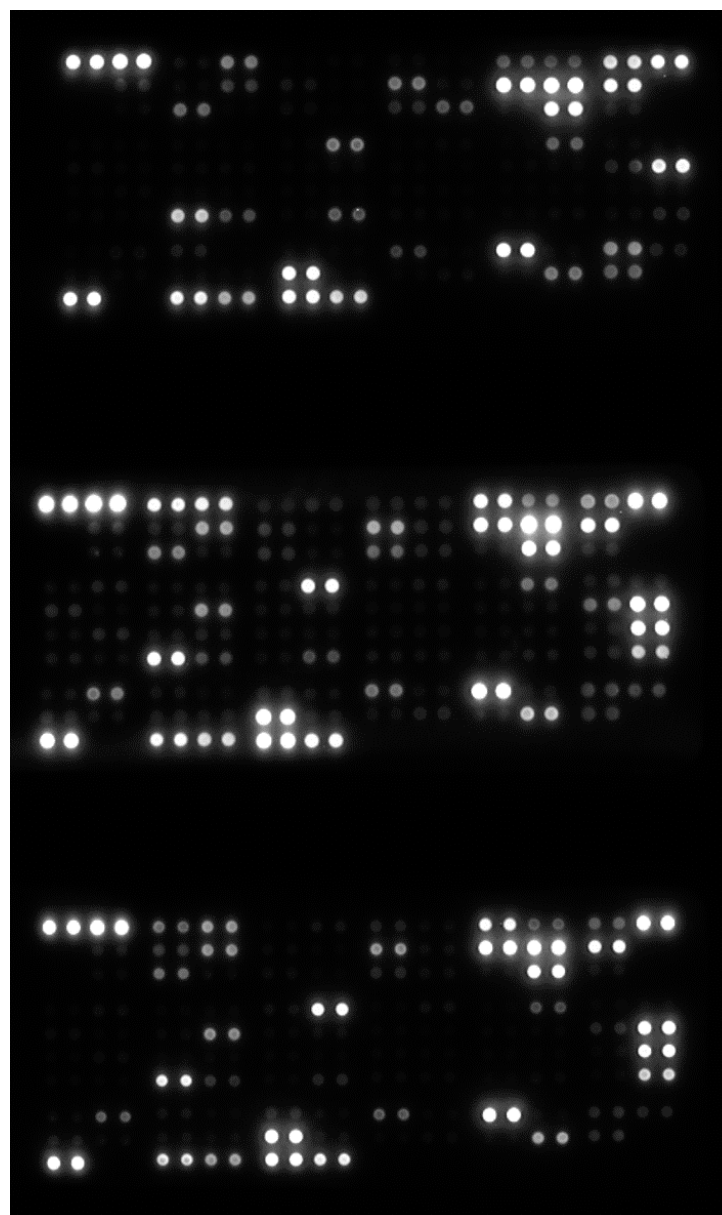

C0135: control one individual male

C0136: CDK pooled male

T0239: CDK pooled female

Supplement: Supplementary file 1 [file jpm-13-00038-s001.zip › Figure S3.pdf]
